# Supplementary material for: Tumor necrosis factor-α (TNF-α) -308G >a promoter polymorphism (rs1800629) promotes Asians in susceptibility to Plasmodium falciparum severe malaria: A meta-analysis
Source: PLoS Negl Trop Dis. 2023 Nov 1;17(11):e0011735. doi: 10.1371/journal.pntd.0011735 (PMC10655976; doi:10.1371/journal.pntd.0011735)
Supplement: S1 Table — (DOCX) [file pntd.0011735.s001.docx]

**S1 Table.** Database search algorithms for *TNF-α* -308G >A polymorphism with susceptibility to severe malaria.

| **Database address** | **Search strings** | | | |
| --- | --- | --- | --- | --- |
| Search as of 15 April 2023 | **1** | **2** | **3** | **4** |
| MEDLINE using PubMed |  |  |  |  |
| <https://www.ncbi.nlm.nih.gov/pubmed/> | 2 | 5 | 4 | 2 |
| Scopus [research article] |  |  |  |  |
| https://www.scopus.com/sources | 88 | 2,029 | 45 | 67 |
| Google Scholar [all in title] |  |  |  |  |
| <https://scholar.google.ca/> | 122 | 9,760 | 402 | 119 |
| Mednar (deep web) |  |  |  |  |
| <http://mednar.com/mednar/desktop/en/search.html> | 145 | 46 | 96 | 155 |
|  |  |  |  |  |

**String search terms**

1. *TNFα-308 G/A* polymorphism and malaria
2. *Tumor necrosis factor-alpha* polymorphism and malaria
3. *rs1800629* polymorphism and malaria
4. *TNFα-308 G/A* polymorphism and severe malaria
