# Supplementary material for: Tumor necrosis factor-α (TNF-α) -308G >a promoter polymorphism (rs1800629) promotes Asians in susceptibility to Plasmodium falciparum severe malaria: A meta-analysis
Source: PLoS Negl Trop Dis. 2023 Nov 1;17(11):e0011735. doi: 10.1371/journal.pntd.0011735 (PMC10655976; doi:10.1371/journal.pntd.0011735)
Supplement: S2 Table — (DOCX) [file pntd.0011735.s002.docx]

**S2 Table.** Summary effects of the *TNF-α* -308G >A polymorphism with susceptibility to severe malaria in Asian HWE-compliant and excluding Asian study with the highest minor allele frequency.

|  |  | Test of association | | | Test of heterogeneity | | | |  |
| --- | --- | --- | --- | --- | --- | --- | --- | --- | --- |
|  | *n* | OR | 95% CI | *P*^a^ | *P*^b^ | *I*^2^ (%) | AM | |  |
| **Overall** |  |  |  |  |  |  | |  | |
| Homozygous | 8 | 1.40 | 0.80-2.45 | 0.24 | 0.40 | 3 | Fixed | |  |
| Recessive | 8 | 1.52 | 0.83-2.79 | 0.26 | 0.50 | 0 | Fixed | |  |
| Dominant | 8 | 1.28 | 0.78-2.10 | 0.33 | 0.00001 | 80 | Random | |  |
| Codominant | 8 | 1.14 | 0.74-1.77 | 0.55 | 0.0001 | 78 | Random | |  |
| **HWE-compliant** |  |  |  |  |  |  |  | |  |
| Homozygous | 7 | 1.31 | 0.73-2.37 | 0.36 | 0.35 | 11 | Fixed | |  |
| Recessive | 7 | 1.32 | 0.73-2.37 | 0.36 | 0.42 | 0 | Fixed | |  |
| Dominant | 7 | 1.11 | 0.68-1.79 | 0.68 | 0.00001 | 78 | Random | |  |
| Codominant | 7 | 1.10 | 0.72-1.68 | 0.67 | 0.0002 | 78 | Random | |  |
| **Asian** |  |  |  |  |  |  |  | |  |
| Homozygous | 5 | 1.93 | 0.75-4.99 | 0.17 | 0.91 | 0 | Fixed | |  |
| Recessive | 5 | 1.82 | 0.71-4.64 | 0.21 | 0.94 | 0 | Fixed | |  |
| Dominant | 5 | **1.95** | **1.06-3.61** | **0.03** | 0.01 | 68 | Random | |  |
| Codominant | 5 | **1.83** | **1.15-2.92** | **0.01** | 0.05 | 58 | Random | |  |
| **Asian HWE-compliant ^†^** |  |  |  |  |  |  |  | |  |
| Homozygous | 4 | 1.73 | 0.58-5.21 | 0.33 | 0.84 | 0 | Fixed | |  |
| Recessive | 4 | 1.68 | 0.56-4.98 | 0.35 | 0.88 | 0 | Fixed | |  |
| Dominant | 4 | 1.64 | 0.86-3.13 | 0.13 | 0.03 | 67 | Random | |  |
| Codominant | 4 | **1.59** | **1.19-2.25** | **0.002** | 0.11 | 51 | Fixed | |  |
| **Asian without highest MAF ^††^** |  |  |  |  |  |  |  | |  |
| Homozygous | 4 | 2.02 | 0.72-5.68 | 0.18 | 0.81 | 0 | Fixed | |  |
| Recessive | 4 | 1.76 | 0.63-4.91 | 0.28 | 0.85 | 0 | Fixed | |  |
| Dominant | 4 | **2.27** | **1.58-3.26** | **0.0001** | 0.19 | 37 | Fixed | |  |
| Codominant | 4 | **2.06** | **1.49-2.85** | **0.0001** | 0.30 | 18 | Fixed | |  |

*TNF-α*: tumor necrosis factor-alpha; *G*: guanine nucleobase; *A*: alanine nucleobase

*n*: number of studies; OR: odds ratio; CI: confidence interval; *P*^a^: *P*-value for association;

HWE: maf: minor allele frequency; HWE: Hardy-Weinberg Equilibrium

*P*^b^: *P*-value for heterogeneity; *I*^2^: measure of variability; AM: analysis model

^†^: excluded study which deviates from HWE, Ubalee et al. [34].

^††^: excluded study which presents the highest MAF, Mohanty et al. [32].

Value in bold indicate statistical significance (*P* < 0.05).
