# Supplementary material for: Tumor necrosis factor-α (TNF-α) -308G >a promoter polymorphism (rs1800629) promotes Asians in susceptibility to Plasmodium falciparum severe malaria: A meta-analysis
Source: PLoS Negl Trop Dis. 2023 Nov 1;17(11):e0011735. doi: 10.1371/journal.pntd.0011735 (PMC10655976; doi:10.1371/journal.pntd.0011735)
Supplement: Attachment — Submitted filename: Response to reviewer TNF Malaria.docx [file pntd.0011735.s005.docx]

**SUMMARY OF RESPONSES TO REVIEWERS’ AND EDITOR’S COMMENTS**

**Reviewers’ Comments**

**Reviewer #1:**

**COMMENT 1**

The conclusions are supported by the data presented. The limitations of the study are well described. However, the authors do not describe how their findings contribute to the understanding and contribution of this polymorphism in complicated malaria among the Asians.

**RESPONSE 1:**

We appreciate the reviewer for taking the time to review our manuscript, and thank you very much for the valuable suggestion. We completely agree with the reviewer that it is valuable information for physicians to be more concerned about severe malaria in Asians than other ethnic groups based on our results.

We explained and added more information regarding the contribution of *TNF-α* -308G>A in complicated malaria among Asians in the revised Discussion section in LINE 459-463 (tracked version MS).

**COMMENT 2**

This is a nice work of meta-analysis and the authors concluded that the polymorphism TNFα -308 is associated with susceptibility among the Asians but not among the Africans. However, my main concern in this study is the minor allele frequency among the Asians. There is a great disparity ranging from 0.04 to 0.26 for the minor allele A among the Asians. Of note, there are two studies from India with completely different minor allele frequency (0.07 and 0.26). It will be interesting for the authors to discuss this disparity and how this could have affected the outcome of the analysis.

**RESPONSE:**

We thank you for the reviewer's concern and suggestion. After excluding Mohanty et al (2019), which showed the highest MAF (0.26) and represented the outlier MAF study from the analysis, it did not affect the main findings, including overall HWE-compliant and Asian subgroup analyses. Significant associations were also found in dominant and codominant models in the Asian subgroup.

However, we provided the results of this analysis in the S2 appendix and explained the part of limitation in the Discussion section in LINE 504-507 (tracked version MS).

……………………………………………………………

**Reviewer #2:** Major and minor comments follow:

**MAJOR**

**COMMENT 1**

Please modify the title, as it is it does not reflect what the authors have studied, since the association with ethnicity is based on the stratification studies, no proper analysis for racial disparities between Africans and Asians were compared.

**RESPONSE 1:**

We appreciate the reviewer for taking the time to review our manuscript, and thank you very much for this suggestion.

We reformulated our manuscript title in the revised MS (tracked version MS).

**COMMENT 2**

It is advised to maintain OR estimates and CIs, leave to one decimal case, more than that is not relevant to understand the effect estimate, and it pollutes the manuscript with numbering.

**RESPONSE 2:**

Thank you for the reviewer’s suggestion. However, we would prefer to retain the value of OR estimates, CIs, and *P*-value to two decimals as it presents a more precise value. For example, 0.71 and 0.76 will become 0.7 and 0.8 when we leave only one decimal. This scenario may distort the exact outcome, although the interpretation was not changed.

However, we formalize all the values in the revised MS to be consistent with two decimals.

**COMMENT 3**

In Methods, line 183, what does criteria vii refers to as duplicated genotype data? Please clarify.

**RESPONSE 3:**

Thank you for this comment. We revised this phrase by adding more explanation in the revised MS in part of the Publication search and selection of studies in the Materials and Methods section in LINE 185-186 (tracked version MS).

**COMMENT 4**

In Methods, line 197, what does corrections for multiplicity refer to? Is it not referring to multiple comparisons’ correction? Please clarify.

**RESPONSE 4:**

Thank you very much for this comment. Yes, corrections for multiplicity refer to multiple comparison corrections. We reformulated the text in the revised MS in part of Data extraction and study quality in the Materials and Methods section in LINE 199-200 (tracked version MS).

**COMMENT 5**

In Methods, line 215, please justify the rationale for applicating the Laplace correction in this scenario? rs1800629 has an overall MAF less than 10%, even reaching to 2% in EAS populations according to ENSEMBL.  Adding a +1 to genotype counts which are probably to AA genotype is a highly sensitive modification in terms of a SNP that already has a low MAF. Also, having 0 genotype counts doesn’t make inviably the performance and calculation neither of the most relevant genetic models (dominant, codominant), or even the OR.

Please justify in detail.

**RESPONSE 5:**

Thank you very much for pointing out this question. We agreed with this reviewer’s concern. Based on several previous studies, the Laplace correction was used in genotype frequency for four genetic models to improve the algorithms' exploring power, although this type of correction may have distorted the finding.

Kido et al (2018) statistical results revealed that risk alleles were enriched in minor alleles, especially for variants with low minor allele frequencies (MAFs < 0.1). In addition, the distribution of observed susceptibility SNPs is skewed toward higher minor-allele frequencies rather than intermediate frequencies compared with SNP allele-frequency distributions in general human populations (Park et al 2011). Our MAF in this study is less than 2%, indicating a low prevalence of mutation probability. For this reason, the Laplace correction in our study has probably less impact on our analysis. Additionally, some literature states this correction is not affected when the variance loss is at the maximum value of 0.5.

With regard to this comment, we added more information in part of the meta-analysis in the Materials and Methods section in LINE 219-221 and part of the strengths and limitations in the Discussion section in LINE 501-504.

**References**

1. Kido et al (2018) Are minor alleles more likely to be risk alleles? BMC Med Genomics. 2018 Jan 19;11(1):3. doi: 10.1186/s12920-018-0322-5.
2. Park et al (2011) Distribution of allele frequencies and effect sizes and their interrelationships for common genetic susceptibility variants. Proc Natl Acad Sci U S A. 2011 Nov 1;108(44):18026-31. doi: 10.1073/pnas.1114759108.

**COMMENT 6**

In Results Line 321, the results from Asians and Africans cannot be compared between them, the results for Africans were not significant, so further description that favour UM lack the basis of statistically significant and should not be interpreted. Please remove.

**RESPONSE 6:**

Thank you very much for this comment. We removed it in the revised manuscript.

**COMMENT 7**

In Results if the study by Ubalee, 2001 deviated in HWE for the control group there should have been a separate analysis in which the study was excluded from summary statistics, as it has been clearly stated how HWE deviations imply in potential genotyping errors and include it in Supplementary material if the association stands after the exclusion of this study.

**RESPONSE 7:**

Thank you very much for the excellent suggestion. We provided additional Supplementary material (Appendix 2) regarding the results of the meta-analysis after excluding the Asian HWE deviation study, Ubalee et al., 2001. From the results, only the codominant model in the Asian subgroup retained a significant association (OR 1.59, 95% CI 1.19-2.25, *P*^a^ = 0.002). We also added and briefly explained these outcomes in part of the meta-analysis outcomes in the Result section in LINE 378-380.

**MINOR**

**COMMENT 8**

In abstract and please review in the rest of the text, when referring to gene and SNP nomenclature modify to TNFA G>A polymorphism or TNFA gene or polymorphism when required.

**RESPONSE 8:**

Thank you very much for this suggestion. We modified gene and SNP nomenclature to *TNF-α* -308G >A throughout the revised manuscript.

**COMMENT 9**

In Introduction, line 151 what does UM stands for?

**RESPONSE 9:**

Thank you very much for this question. UM stands for uncomplicated malaria. We changed to the full name in the revised manuscript.

**COMMENT 10**

In Materials and Methods please modify the subtitle (line 164) Case-control definition and remove lines 167 -169 that indicate that the meta-analysis followed PRISMA guidelines to include them as the initial line for Publication Search and Study selection in line 172.

**RESPONSE 10:**

Thank you very much for this suggestion. We completely revised the manuscript regarding the reviewer’s recommendation.

**COMMENT 11**

Minor Typing and concordance in English redaction errors should be revised. E.g. line 221 in methods correct for heterogeneity.

**RESPONSE 11:**

Thank you very much for this comment**.** We completely revised the manuscript regarding reviewer’s notion.

**COMMENT 12**

Line 456. Remove bunch and change for other term.

**RESPONSE 12:**

Thank you very much for this comment. We substituted many meta-analysis procedures for various ones in the revised MS.

**COMMENT 13**

Typo is shown in result's part of manuscript (line 271, page 12): "...... the statistical power ranged from 18.7 to 80.1% , in which .................." should be "...... the statistical power ranged from 12.4 to 80.1% , in which .................." .

**RESPONSE 13:**

Thank you very much for the reviewer’s pointing out this error. We thus changed to the correct one in the revised manuscript.

……………………………………………………………

**Reviewer #3:**

**COMMENT 1**

This manuscript has a good composition, harmonization and clarification of writing. Value of meta-analysis of to this study is the usefulness of the association of TNF alpha gene polymorphism and severity of malaria in Asian population. It is valuable information for physician to have more concerned about severe malaria in Asian than other ethnic group.

**RESPONSE 1:** We appreciate the reviewer for taking the time to review our manuscript, and thank you very much for your appreciation of our manuscript.

……………………………………………………………

**Editor’s Comments**

1. Figure Files:

While revising your submission, please upload your figure files to the Preflight Analysis and Conversion Engine (PACE) digital diagnostic tool, https://pacev2.apexcovantage.com. PACE helps ensure that figures meet PLOS requirements. To use PACE, you must first register as a user. Then, login and navigate to the UPLOAD tab, where you will find detailed instructions on how to use the tool. If you encounter any issues or have any questions when using PACE, please email us at [figures@plos.org.](mailto:figures@plos.org.)

 RESPONSE: We have uploaded Fig 1 and Fig 2 files to the PACE digital diagnostic.

2. Data Requirements:

Please note that, as a condition of publication, PLOS' data policy requires that you make available all data used to draw the conclusions outlined in your manuscript. Data must be deposited in an appropriate repository, included within the body of the manuscript, or uploaded as supporting information. This includes all numerical values that were used to generate graphs, histograms etc.. For an example see here: http://www.plosbiology.org/article/info%3Adoi%2F10.1371%2Fjournal.pbio.1001908#s5.

 RESPONSE: Since the article is a systematic review, no data are available for this purpose.

3. Reproducibility:

To enhance the reproducibility of your results, we recommend that you deposit your laboratory protocols in protocols.io, where a protocol can be assigned its own identifier (DOI) such that it can be cited independently in the future. Additionally, PLOS ONE offers an option to publish peer-reviewed clinical study protocols. Read more information on sharing protocols at <https://plos.org/protocols?utm_medium=editorial-email&utm_source=authorletters&utm_campaign=protocols>

 RESPONSE: Since the article is a systematic review, no laboratory protocol is involved in the study.
